# A Conversation with James LaBelle, MD, PhD, Associate Professor of Pediatric Hematology/Oncology/Stem Cell Transplant, University of Chicago

**DOI:** 10.1017/cts.2024.628

**Published:** 2024-11-08

**Authors:** Clinical Research Forum

**Affiliations:** Clinical Research Forum, Washington DC, USA

## Top 10 Clinical Research Achievement Awards Q & A

This article is part of a series of interviews with recipients of Clinical Research Forum’s Top 10 Clinical Research Achievement Awards. This interview is with James LaBelle, MD, PhD, Associate Professor of Pediatric Hematology/Oncology/Stem Cell Transplant, University of Chicago. Dr. LaBelle provides care for children of all ages with cancer and blood diseases, and he specializes in stem cell transplantation. Dr. LaBelle received a 2024 Top 10 Clinical Research Achievement Award for Stem Cell Gene Therapy for Patients with Sickle Cell Disease. *The interview has been edited for length and clarity.*

## How did you get interested in a career in clinical research?

Ever since I was a child, I have been fascinated by how things work, and I always knew I wanted to do science, although I didn’t know what kind of science I wanted to do. One of my biggest influences back then was “Mr. Wizard’s World” with Don Herbert. It was a children’s television show devoted to science and technology, and it was so impactful that I actually used it as the subject of my essay on my medical school application. At first, while in college, I thought I would be a plant physiologist and maybe build a bigger tomato. But then I did a summer research fellowship at the Mayo Clinic in Minnesota, and that’s when the “gene” switched on for going into medicine.

## What was it that flipped the switch?

I spent that summer in a lab working on human immunology and I just got hooked. By the end of the fellowship, I knew I wanted to do both medicine and clinical research, and I went on to a combined MD PhD program at the Medical College of Wisconsin in Milwaukee. While there, I worked with Bob Truitt, one of the pioneers in bone marrow transplantation, and he was a huge inspiration to me. That was the start of my journey to become a stem cell transplant physician.

## How did pediatrics fit in?

I knew very early based on my prior training that I wanted to do stem cell transplantation and I focused clinically on bone marrow transplantation in children and young adults. I did my pediatric residency at Boston Children’s Hospital, and then my pediatric hematology oncology fellowship at the Dana Farber Cancer Institute. I was also working in the lab of Loren Walensky, MD, PhD, who is a pediatric hematology/oncology physician, and that experience was very rewarding and influential. I then went to the University of Chicago and met Dr. John Cunningham, a leader in the field of pediatric stem cell transplantation. Working with him helped me hone my clinical skills, and now I’m the director of our pediatric stem cell transplantation program. Clinically, I concentrate on stem cell and cellular therapies for children, adolescents, and young adults for malignant and nonmalignant conditions. That’s how I got involved with the award-winning study.

## What were the findings of the award-winning research?

Sickle cell disease is an autosomal recessive disorder caused by mutations in the gene *HBB*, which encodes the β-globin subunit of adult hemoglobin. Symptoms of sickle cell disease appear during infancy as γ-globin gene (*HBG1* and *HBG2*) transcription switches to *HBB*, causing a shift from fetal hemoglobin to adult hemoglobin in red cells. With this research we were able to show that CRISPR-Cas9 disruption of a negative regulatory region in *HBG1* and *HBG2* promoters of autologous hematopoietic stem cells (HSCs) obtained from participants with sickle cell disease resulted in induction of red-cell fetal hemoglobin and a partial correction of sickle cell disease.

## Why is this paper so important?

It’s important for a few different reasons. First, this is the first report on CRISPR-Cas9-mediated gene editing of the promoter regions of hemoglobin. We tested a unique hypothesis, and it worked. That opens the door to new therapies for not only sickle cell, but other diseases, as well. What we did as a group reflects a road map for how the bench goes to the bedside. Another important aspect of this work is that the stem cells we used were cryopreserved (frozen) on site, at each center. Previous studies of autologous hematopoietic stem cell transplantation for sickle cell used freshly collected cells to manufacture the cellular drug product. But being able to cryopreserve the stem cells means that down the road, this therapy could theoretically be available to centers that are not as highly resourced as large academic institutions as coordinated shipment of products is less complicated.

## These are major milestones in more than 100 years of sickle cell research

Yes, this particular study stands on the shoulders of true medical giants. That includes those who discovered the genetic and molecular basis of sickle cell disease and those who figured out the mechanism for the change from the fetal hemoglobin state to the adult hemoglobin state. The first western description of sickle cell disease occurred in 1910, in Chicago by the way, and then it took decades more, until 1972, for the National Sickle Cell Anemia Control Act, which created the first federal programs for sickle cell education, counseling, research, treatment, and screening. After that, the discoveries about the molecular basis of sickle cell disease rapidly advanced, but we weren’t able to actually target it – until now. It’s really exciting to know that we may now have the potential for transformative and durable treatments for sickle cell and other similar diseases.

## What’s next? Where does this research go from here?

This is just the beginning. Because of this research, the door is now open for even more advancements. Gene therapy has had starts and sputters for a long time and that’s mostly associated with the packaging system we’ve been using to administer it. But as we learn more about integration and how to use CRISPR-Cas9 to edit genes, we’re going to start seeing much more progress across a variety of diseases.

## How do you balance research with your clinical work?

I have a lab dedicated to drugging the the BCL-2 family of apoptotic proteins in immune cells, and we also do nanotechnology for drug delivery of compounds to reactivate cell death in cancer cells. That’s somewhat, but not completely, related to what I do clinically, and honestly, balancing the two takes a little bit of academic ADHD [attention-deficit/hyperactivity disorder]. As I was starting my career, I had lots of irons in the fire, in the hopes that some of them would take off. That was my approach, for good or for bad, and it benefited me personally because that’s just how my brain operates. But what started as maybe 10 irons in the fire is now three irons in the fire and each one is burning much hotter. I still enjoy the variety. It’s just more manageable now.

## What about the variety is so appealing?

I think if I just did clinical medicine all the time, I would get a little burnt out. For me, clinical research is really the cure for career burnout. It allows you to ask questions and to probe deeply. I got into this job because of my innate curiosity about the way things work and how I could use that to help people feel better. That’s why it’s very fulfilling to do both, to have time for research and asking questions, as well as clinical medicine.

## What do you do outside of work that motivates you?

I’m married and have a family – a 16-year-old, a 13-year-old, and a 10-year-old – so that keeps me busy. Other than that, I love being outside. For me, gardening is like free therapy. I’m also a fan of baseball and hockey – so much so that three years ago I took up playing hockey, even though I had never skated before. Now I play on two teams, and it’s fantastic. It’s humbling to learn a new sport as an adult and then try to get better at it, but it’s also really invigorating. All of these things are important in their own way and they help me keep my perspective.

